# Dynamics of the association between visual and auditory functional changes in glaucoma. Preliminary results

**DOI:** 10.22336/rjo.2023.22

**Published:** 2023

**Authors:** Alina Mihaela Neacșu, Nicoleta Anton, Lucian Lăpușneanu, Ovidiu Mușat, Maria Cristina Andrei, Mihaela Coșman, Nicoleta Andreea Țovănac, Dumitru Ferechide

**Affiliations:** * Department of Ophthalmology, Clinical County Emergency Hospital, Brăila, Romania; ** Department of Ophthalmology, “Grigore T. Popa” University of Medicine and Pharmacy, Iaşi, Romania; *** Department of Otolaryngology, Clinical County Emergency Hospital, Brăila, Romania; **** Department of Ophthalmology, “Dr. Carol Davila” Central Military Emergency Hospital, Bucharest, Romania; ***** Department of Radioimaging, Clinical County Emergency Hospital, Brăila, Romania; ****** Department of Neurosurgery, Clinical County Emergency Hospital, Brăila, Romania; ******* Department of Neurology, Clinical County Emergency Hospital, Brăila, Romania; ******** Physiology Department, “Carol Davila” University of Medicine and Pharmacy, Bucharest, Romania

**Keywords:** hearing loss, pure tone audiometry (PTA), visual field parameters, MD, Cal Hov, slope profile

## Abstract

Glaucoma is a multifactorial degenerative optic neuropathy characterized by the irreversible loss of retinal ganglion cells. Vascular, genetic, anatomical and immune factors are present in etiopathogenic mechanisms. Being the second cause of blindness worldwide after cataract, and with an irreversible character, glaucoma has turned into a disease with a significant impact on public health. Patients with primary open-angle glaucoma (POAG) may have central neurodegenerative changes, such as sensorineural hearing loss and static changes.

**Aim:** The aim of this study was to estimate the connections between visual and auditory functional changes in glaucoma. The subjects were grouped as follows: patients with glaucoma compared with patients without glaucoma, while trying to identify the functional defect of the optic nerve (visual field) and a hearing testing (audiogram).

**Materials and methods:** The prospective, cross-sectional study included 32 eyes of 16 subjects with POAG in a group of study and 24 eyes of 12 healthy subjects in the other group, with a mean age of both groups between 61,64 ± 6,53 years old. Both groups were examined from ophthalmological, audiological and radioimaging perspectives with Pure-tone audiometry (PTA) and magnetic resonance imaging (MRI) for brain. All patients had ophthalmologic assessments according to a standardized protocol. Moreover, auditory functional parameters (audiometry) were recorded.

**Results:** Female cases, over 65 years old, with residency in a city, predominated in the group of study. Compared to the control group, patients in the group of study had average levels of the PTA and modified visual field (VF) parameters. Multivariate analysis demonstrated that the correlation of PTA was indirect, reduced in intensity, both with MD (r = -0.108; p = 0.585), Cal HOV (r = -0.268; p = 0.168) and the slope profile of the right eye. Multivariate analysis demonstrated that there was a correlation of the right PTA, which was indirect, reduced in intensity, both with MD (r = -0.108; p = 0.585), Cal HOV (r = -0.268; p = 0.168) and the slope profile of the right eye. The left ear PTA correlation was indirect, moderate in intensity, statistically significant with both MD (r = -0.584; p = 0.001) and slope profile (r = -0.377; p = 0.048) and reduced as intensity with Cal HOV (r = -0.147; p = 0.456) of the left eye.

**Conclusions:** Patients with POAG showed changes in audiometry in connection with ophthalmological parameters, a fact suggesting that the auditory system might have been affected in POAG. This study highlighted the interdisciplinarity of the medical field with the aim of ensuring the quality of life of glaucoma patients. A good collaboration between the ophthalmologist and the otolaryngologist was very important for our patients.

**Abbreviations: **RE = Right eye, LE = Left Eye, POAG = Primitive Open Angle Glaucoma, PTA = Pure-tone audiometry, VF = visual field, MRI = magnetic resonance imaging, IOP = Intraocular pressure, BVAC = best visual acuity corrected, MD = mean defect, CNS = central nervous system, SAP = standard automated perimetry, CCT = central corneal thickness, ONH = Optic nerve head, CDR = Cup/ Disc ratio, RNFL = Retinal fiber layers

## Introduction

Glaucoma is a multifactorial and degenerative optic neuropathy characterized by the irreversible loss of retinal ganglion cells. Vascular, genetic, anatomical and immune factors are involved in etiopathogenic mechanisms. Glaucoma is a significant public health problem, being the second leading cause of blindness in the world after cataract, but, which is irreversible.

People aged over 60 years with pathological heredo-collateral antecedents of glaucoma, steroid users, diabetics, patients with high myopia, high blood pressure, central corneal thickness <5 mm, and eye injuries, are at an increased risk of glaucoma [**1**].

In 2020, approximately 76 million people suffered from glaucoma and their number is expected to grow to 111.8 million by 2040 [**1**].

The sharp increase in the number of existing and estimated patients, as well as their degree of visual impairment that significantly affects the quality of life, justify the scientific steps for an as early as possible diagnosis to choose the appropriate treatment with the main aim of preserving sight with social integration.

Visual impairment affects physical functioning, while hearing impairment greatly affects the social functioning [**2**]. Dual sensory impairment, hearing and vision, compromises the two main ways of communication, listening and reading speech, and can determinate social isolation [**3**,**4**], which can affect the quality of life. The double sensory loss affects the cognitive function greater than the unilateral loss of vision or hearing [**5**-**9**], the two functions being interconnected cerebrally. In perspective, the aging of population will be associated with the prevalence of glaucoma that will increase, and the loss of the two senses will become more frequent, the two functions being functionally interconnected [**10**].

The aim of this preliminary study, which is part of a complex evaluation study of glaucoma patients, was to establish the possible correlations between the functional visual and auditory impairment in glaucoma, elements that can improve the diagnostic and treatment algorithm. The current study refers to glaucoma patients, whose disease is not advanced, and tries to identify elements that bring benefit to the early diagnosis of the glaucomatous disease.

The visual field is a very important element in the diagnosis and evaluation of the patient with glaucoma. From the indicators offered by the SAP (standard automated perimetry), we considered the following in the study: Cal HOV, slope profile, and MD.

The Cal HOV level is a fixed sensitivity level in four calibration locations placed on a 10° ring, after omitting two extreme values and calculating the average figure from the other two. This value is a HOV value, calculated during calibration.

Slope profile or the slope of the perimeter profile is a quantitative index that contains information about how smoothly or steeply the light sensitivity lows from the center to the periphery in dB/ 10°. For screening, it is set to 3 (dB/ 10°) by default.

MD (mean defect) specifies the loss of ocular sensitivity and is the average difference between the “ideal” profile calculated by the computer for a given patient based on the results of his evaluation and appropriate age norm. The MD calculation is the average value of the locations on the Total Deviation graph – for 24, 30 fields and from the Age deviation graph - for radial maps. The MD index shows how far the patient’s visual field has deviated from normal values. For below-normal vision, the MD index is negative [**11**,**12**].

The auditory and visual functions are fundamental factors in the cognitive processes that ensure the integration of the individual in society through complex mechanisms. The auditory functional evaluation and the management of hearing loss are very important for all the patients, regardless of their age, to ensure the safety of life.

Audiometry is a way of evaluating hearing thresholds. The audiogram evaluates and records graphically the type of hearing loss (sensorineural, conductive or mixed). Several etiological situations that generate hearing changes develop characteristic patterns on the audiogram. Audiograms are the golden standard evaluation for the identification of hearing deficits. The audiogram is a functional test that measures both conduction air and bone. Air conduction assesses the transmission of sound through the outer, middle, and inner ear to the central nervous system (CNS) for auditory integration. Bone conduction represents the transmission from the inner ear only to the CNS [**13**,**14**].

Since the 1920s, threshold audiometric data (pure tone testing) have been used clinically to classify the degree and type of hearing loss. Audiometry uses different intensities of sound emitted over a range of frequencies to determine possible hearing loss, and the results are represented graphically as an audiogram. A typical audiogram shows thresholds at frequencies over a slightly wider range than human speech: 250, 500, 1000, 2000, 3000, 4000, 6000, and 8000 Hz. Primary speech frequencies range from 500 to 4000 Hz, although normal human hearing works from ~20 Hz to ~20,000 Hz [**14**]. PTA, “pure tone average”, is the arithmetic mean of air conduction thresholds at 500, 1000, and 2000 Hz, which is calculated for each patient. However, PTA does not inform about high-frequency hearing loss. For each frequency, PTA is a graphic representation, in which the X-axis represents the frequency of the sound in hertz and the Y-axis represents the (inverse) sound intensity in decibels. ASHA (American Speech-Language-Hearing Association) set standardized levels for hearing loss.

The auditory pathway has the auditory cortex as its central projection, which is primary located in the superotemporal region of the gyrus superior temporal with extension on the extern surface of the temporal lobe, on an extension of the insular cortex and included in the lateral region of the parietal operculum. For association, the auditory cortex receives information both from the primary cortex, the thalamic areas located adjacent to the medial geniculate bodies, but also from other visual areas located adjacent to the primary area. The primary somatosensory areas, which are areas of association that receive information both from the primary and secondary areas, from the subthalamic structures necessary for the complex integration of information, are located at the cortical level, additionally to the primary and secondary motor cortical areas. Among these association areas, the parieto-occipital-temporal has a great role. Wernicke’s area, located at this level, is involved in understanding language. Behind this, is the area for language read processing at the level of the angular gyrus, in the lateral side of the occipital lobe. At this level, numerous areas of association with the auditory, visual analyzer with major implication in thought and language processes, are located. The face recognition area is located on the medial face of the occipital and parietal lobes. All the somatic, auditory and visual association areas forms are complex mechanisms in the interpretation of sensory experiences and send information to the posterosuperior Wernicke’s area in the temporal lobe, which informs Broca’s language area located in the frontal area (complex integrative systems) [**15**].

## Materials and Methods

*Study design*

A prospective, cross-sectional, observational study took place from October 2021 to December 2022 in the Ophthalmology Department of Clinical County Emergency Hospital of Brăila. Patients were evaluated for a period of 6 months after enrollment. The Ethics Committee of Clinical County Emergency Hospital of Brăila approved the study (no.1/ 3.09.2021) in conformity with the deontological and ethical rules for medical and research practice, in accordance with the Declaration of Helsinki and informed consent forms were obtained from the patients. 

*Inclusion criteria*

The inclusion criteria were the following: glaucoma disease (high intraocular pressure without treatment, visual field defects on SAP, optic nerve head morphology (rim loss and corresponding retinal nerve fiber layer and visual field loss), open iridocorneal angle, both eyes affected, age range between 47 to 70 years, refractive error before 4 diopter sphere and a 2-diopter cylinder, and transparent ocular media. All participants with glaucoma were on topical glaucoma medications. A history of uneventful cataract surgery was allowed for study participants. The volunteers needed to have an intraocular eye pressure less 21 mmHg in both eyes, normal optic discs and normal SAP, and no ocular or systemic diseases that could affect optic nerve structure or visual function.

*Exclusion criteria*

The exclusion criteria were of two types: ophthalmological and hearing.

Ophthalmological exclusion criteria referred to systemic and ocular diseases known to affect visual and hearing processing for each participant in study. These criteria included best-corrected visual acuity (BCVA) worse than 20/ 100, exceeded refractive error ± 4 sphere diopters or 2-cylinder diopters, cataract, close angle, occludable angle, angle anomalies, anterior peripheral synechiae, hyperpigmentation, exfoliating material. We excluded inflammatory or retinal eye diseases, previous eye trauma, diabetic retinopathy, neuro-ophthalmological diseases, tilted disc, drusen or systemic diseases capable of causing visual field loss or optic nerve damage, intracranial injury or orbital diseases. Moreover, all the participants with poor cooperation or claustrophobia were excluded from the study. Hearing exclusion criteria were the following: conductive and family history of hearing loss, diseases of the neck muscles, high noise exposure, ear infection, tympanic membrane perforation, ear surgery, head trauma, active upper respiratory tract infection, history of ototoxic drugs and systemic diseases that affected hearing, like auditory trauma and chronic lesions of the auditory apparatus. All the participants without other neurological diseases that could have disturbed the functional investigation were included in the study.

The subjects included in the study were grouped as follows: a group with glaucoma that was compared to a group without glaucoma only, as well as the identification of the evolution of structural changes in the optic nerve in patients with glaucoma and in normal patients. The study was prospective and cross-sectional, and was performed on 32 eyes of 16 subjects with POAG in a study group and 24 eyes of 12 healthy subjects in the control group, with a mean age of 62 years in both groups. All 28 participants were enrolled from the Ophthalmology Department of Clinical County Emergency Hospital of Brăila. Of the 16 patients in the study group, 11 were females and 5 were males and of the 12 healthy volunteers in the control group, 9 were females and 3 were males, with a mean age of 62 years. Initially, the study group started with 33 patients with glaucoma, but based on the rigorous and restrictive inclusion and exclusion criteria explained in the study design, 9 patients were excluded due to the severity of the glaucomatous disease. Out of the rest, 8 patients were excluded because they did not meet the stated criteria and thus, only 16 patients with glaucoma were included in the study. The control group included 12 volunteers, because all these patients had to meet the rigorous inclusion and exclusion criteria. It should be mentioned that all participants were examined by MRI, a difficult investigation to accept especially for healthy subjects.

*Evaluation protocol*

*Ocular examinations*

All participants were subjected to a detailed medical history and ocular examinations: BCVA, refraction, intraocular pressure (IOP), slit-lamp and fundus examinations, and central corneal thickness (CCT). Refractive errors were evaluated by an auto refracto-keratometer (Reichert RK600, Reichert Technologies Inc, NY, USA). Bilateral IOP was measured once in the morning between 10:00 AM with Goldmann applanation tonometry and anterior segment was evaluated by slit-lamp (Reichert Xcel 200, Reichert Technologies Inc, NY, USA) by ophthalmologists. CCT was measured with central corneal ultrasonic pachymetry (OcuScan RxP; Alcon Laboratories Inc., Irvine, CA), and 90D lens Volk Optical USA were used for fundus examinations. 3 mirror lens Ocular Instruments, USA were used for anterior chamber angle. Visual field (Standard Automatic Perimetry - SAP) and OCT were performed. Visual field refers to at least two reliable SAP (Optopol Technology PTS 900, Glaucoma field, Strategy Fast threshold, Optopol Technology S.A., Zawiercie, Poland). We reexamined the patients with more frequent breaks, if they were tired, or with better training, if false negative errors were higher than 15% or false-positive or false-negative rates were higher than 15%. Defects of SAP results were defined as typical glaucomatous, with integrated perimetric defect in the clinical context, MD (mean defect) and pattern defect (PD). Spectral-domain optical coherence tomography (OCT) imaging was performed using Optopol SOCT Copernicus (version 4.20, rev.5, SOCT Software 4, 3, Optopol Technology S.A., Zawiercie, Poland). The images were obtained under pupil dilation using the ultra-high-resolution spectral-SD OCT Copernicus, to acquire tomograms with a wavelength of 850 nm and a theoretic axial resolution of 5.0 μm (7×7×2 mm, 75 B-scans, 743 A-scans per B-scan, fixation of target set to image the optic disc). The cup diameter (using a cup offset 150 μm anteriorly to the disc axis), cup maximal depth, and horizontal rim size (distance between horizontal disc and cup diameters) and temporal and nasal height (between the horizontal cup diameter level and the RNFL, limited on the periphery by the disc margins) were measured automatically. Good-quality scans, with a signal strength of at least 7, without RNFL discontinuity, involuntary saccade, blinking artifacts, were used. For patient comfort, OCT was performed in a quiet and dark room, and artificial tears were used before OCT image acquisition. Optic nerve assessment included the evaluation of the optic disc size, neuroretinal rim shape, rim area, disc area, area Cup/ Disc ratio, average vertical CDR and a retinal nerve fiber layer in all sectors.

*Imaging evaluations*

All the participants underwent brain magnetic resonance imaging (MRI) to exclude other ear diseases like cochlear ossification, active mastoiditis or cerebral tumor. MRI was performed with General Electric Healthcare MRI Signa Explorer, USA, 2019; 1,5 Tesla with the orbit and head protocol was performed by a specialist radiologist. All the subjects underwent an examination of the head first, in supine position. This device allows head stabilization with foam cushions on both sides to minimize head motion. To minimize eye movement and possible anatomical changes during image acquisition process, participants were advised to look straight ahead to ensure standardization and repeatability of the MRI scans. Orbit and head tests were combined for the MRI scans of the optic nerve and cerebral tissue.

*Ears examinations*

A specialist otolaryngologist examined all the participants, who underwent bilateral ear examination prior to hearing testing with Heine beta 200 otoscope. Audiometry evaluation was performed using pure-tone audiometry in a special double-walled soundproof booth quit room. Air-condition pure-tone thresholds audiometry was performed using the MAICO MA53 Audiometer, two channels (MAICO Diagnostic GmbH, Salzufer 13/ 14, D 10587 BERLIN), Noah 9 software, or each ear at six frequencies (125, 250, 500, 1000, 2000, 4000, and 8000 Hz), by a well-trained examiner, and the results were recorded. Hearing loss was a pure-tone average threshold of >40 dB at 125, 250, 500, 1000, 2000, 4000 and 8000 Hz, averaged for both ears. Hearing impairment was mild or moderate-to-profound. ASHA recommendations thresholds classify normal hearing at 0-25 dB (decibels), mild hearing loss at 26-40 dB, moderate hearing loss at 41-55 dB, moderate-severe hearing loss at 56-70 dB, severe hearing loss at 70-90 dB, and a profound hearing loss at more than 90 dB a [**16**,**17**]. If initial testing was deemed unreliable, audiometry testing was repeated.

*Statistical Analysis*

The statistical functions of SPSS version 18.0 software (SPSS Inc, Chicago, IL) were used for the data that were loaded and processed. The analysis of the intra- and intergroup dispersion of the dependent variable was performed with ANOVA test. When the examined variable had continuous values, the coefficient of variation (CV%) was used, which highlighted the percentage deviation between two averages, providing results on the homogeneity of the series of values, and the Skewness test (-2 < p < 2) validated the normality of the series of values. The following quantitative variables were applied for the calculation of the significant difference between two or more groups, depending on the distribution of the value series, at the significance threshold of 95%: the t-Student test, which is a parametric test that compares the mean values recorded in 2 groups with normal distributions; the F-test (ANOVA), which is used when comparing 3 or more groups with normal distributions, combined with the application of the Bonferroni correction (Bonferroni post-hoc) to reduce the error rate when testing several hypotheses; the c2 test, which is a non-parametric test that compares 2 or more frequency distributions from the same population, and it is applied when the expected events are excluded; the Kruskal-Wallis test, which is a non-parametric test that compares 3 or more frequency distributions between groups; the “Pearson” correlation coefficient (r), which represents the correlation of 2 variables from the same group (direct/ indirect correlation). The study of the correlation between different parameters was carried out with by employing the Pearson correlation coefficient, which reproduces the intensity of the statistical links and their meaning. The values of the correlation coefficient were between [-1, +1]: if the correlation coefficient tended to +1 (direct correlation), or to -1 (indirect correlation), there was a strong linear dependence of the parameters; the closer the correlation coefficient was to 0, the lower the intensity of the link. Multiple linear regression aims to highlight the relationship between a dependent variable (explained, endogenous, result) and a set of independent variables (explanatory, factorial, exogenous, predictors).

## Results

*Epidemiologic characteristics*

*Gender distribution*

**Fig. 1:**
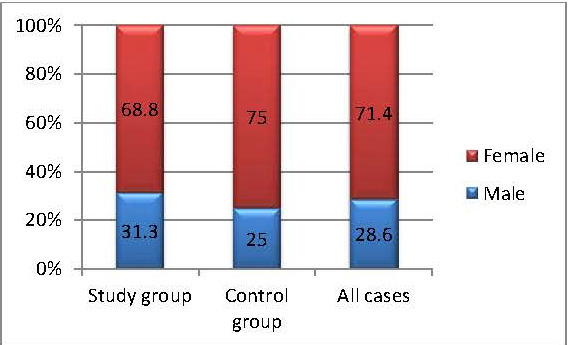
The structure of the groups by gender

In the study/ control group, female patients predominated (71.4%), the F/ M ratio being 2.5/ 1. The percentage distribution of both groups was homogeneous (68.8% vs. 75%; p=0.716) (**Fig. 1**).

*Age distribution*

**Fig. 2:**
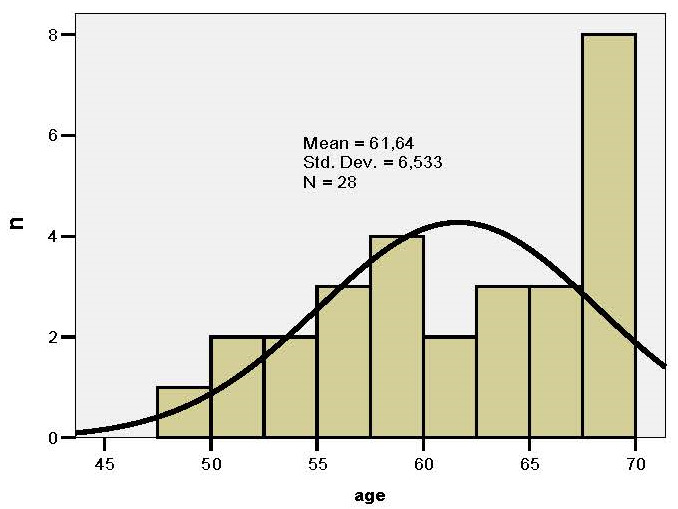
Histogram of age

The series of values for age were homogeneous, which suggested that the tests of statistical significance could be applied (**Fig. 2**): the average age in the study group was significantly higher compared to the control group (63.69 vs. 58.92 years; p=0.05).

*Origin distribution*

**Fig. 3:**
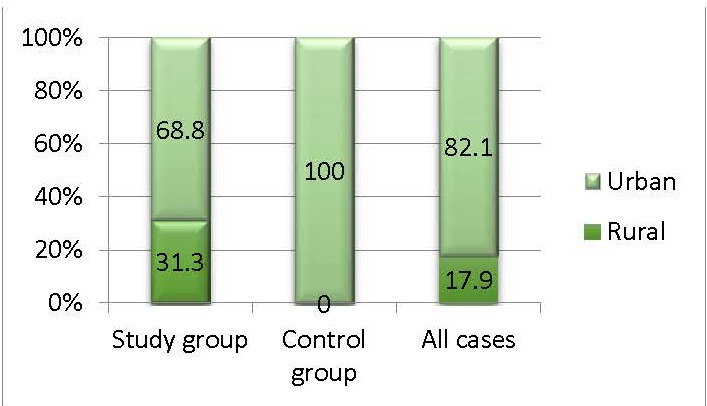
The structure of the groups by residence

Participants from the city predominated (urban) (82.1%), the U/ R ratio being 4.6/ 1 in the study/ control group. It was observed that all the patients in the control group and 68.8% of the study group came from the urban environment (p=0.011) (**Fig. 3**).

*Educational status distribution*

**Fig. 4:**
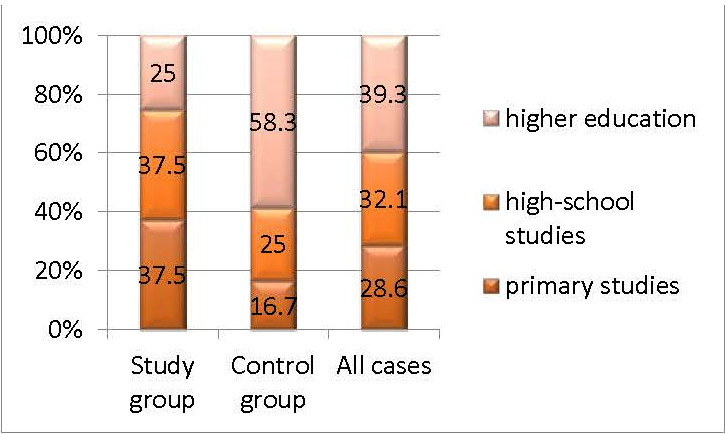
The structure of the groups by education

In the case study presented, more than 70% of the patients had secondary education (32.1%) or higher education (39.3%). It was noted that over 58% of the patients in the control group and 25% in the study group had higher education (p=0.05) (**Fig. 4**).

*Clinical parameters*

*Pure tone audiometry (PTA)*

Because PTA was a homogeneous variable in both the right and the left ear, we were able to apply statistical significance tests (**Table 1**).

- Right ear PTA varied from 0 to 30 dB, the average level being 16.21 dB ± 7.14, close to the median value (15 dB), and the result of the Skewness test = 0.441;

- Left ear PTA varied from 5 to 52 dB, the average level being 19.29 dB ± 10.87, close to the median value (18 dB), and the Skewness test result = 1.292 (without statistical significance).

**Table 1:** Descriptive data PTA (dB)

		right	left
N		28	28
Average		16,21	19,29
Median		15	18
Standard Deviation		7,14	10,87
Alternative		44,05	56,35
Skewness Test		0,056	1,292
Er. standard Skewness		0,441	0,441
Minimum		0	5
Maximum		30	52
Percentiles	25	11	12
	50	15	18
	75	22	25

*VF parameters: MD, Cal HOV, slope profile*

Because VF was a homogeneous variable in both the right and the left eye, we were able to apply statistical significance tests. After these analyses, we were able to state that the data were in accordance with the grading of the glaucomatous disease.

*MD*: Comparative MD by study groups (dB) (**Table 2**):

- in the study group, the MD in the RE varied from -0,44 to 0,30, the average level being -0,156 ± 0,201, close to the median value (-0,210 ), and the result of the Skewness test = 0.949;

- in the study group, the MD in the LE varied from -0,59 to 0,31, the average level being -0,186 ± 0,246, close to the median value (-0,230), and the result of the Skewness test = 0.564;

- in the control group, the MD in the RE varied from -0,10 to 0,07, the average level being -0,025 ± 0,06, close to the median value (-0,035), and the result of the Skewness test = 0.326;

- in the control group, the MD in the LE varied from -0,16 to 0,10, the average level being -0,003 ± 0,08, close to the median value (-0,010 ), and the result of the Skewness test = -0,468;

**Table 2:** Comparative MD by study groups (dB)

	RE MD			LE MD		
	Study Group	Control Lot	All groups	Study Group	Control Lot	All groups
N	16	12	28	16	12	28
Average	-0,156	-0,025 b)	-0,100	-0,186	-0,003 b)	-0,108
Median	-0,210	-0,035	-0,085	-0,230	0,010	-0,065
Std. Dev	0,201	0,06	0,168	0,246	0,08	0,212
Alternative	0,040	0,004	0,028	0,061	0,006	0,045
Skewness Test	0,949	0,326	0,092	0,422	-0,468	-0,444
Er. std. Skewness	0,564	0,637	0,441	0,564	0,637	0,441
Minimum	-0,44	-0,10	-0,44	-0,59	-0,16	-0,59
Maximum	0,30	0,07	0,30	0,31	0,10	0,31
Percentiles 25	-0,285	-0,083	-0,260	-0,325	-0,068	-0,270
50	-0,210	-0,035	-0,085	-0,230	0,010	-0,066
75	-0,065	0,035	-0,023	-0,045	0,070	0,038
a) p<0,001 b) p<0,05 ns) p> 0,05						

*Cal HOV*: Comparative Cal HOV by study groups (dB) (**Table 3**):

- in the study group, the Cal HOV in the RE varied from 18 to 21, the average level being 20,44 ± 1,209, close to the median value 21, and the result of the Skewness test = 0.564;

- in the study group, the Cal HOV in the LE varied from 18 to 21, the average level being 20,06 ± 1,784, close to the median value 21, and the result of the Skewness test = 0.637;

- in the control group, the Cal HOV in the RE varied from 18 to 24, the average level being 20,67 ± 2,015, close to the median value 21, and the result of the Skewness test = 0.637;

- in the control group, the Cal HOV in the LE varied from 18 to 24, the average level being 20,05 ± 1,784, close to the median value 21, and the result of the Skewness test = 0,441;

**Table 3:** Comparative Cal HOV by study groups (dB)

	RE Cal HOV			LE Cal HOV		
	Study Group	Control Lot	All groups	Study Group	Control Lot	All groups
N	16	12	28	16	12	28
Average	20,44	20,67 ns)	20,54	20,06	20,50 ns)	20,25
Median	21,00	21,00	21,00	21,00	21,00	21,00
Std. Dev	1,209	2,015	1,575	1,436	1,784	1,578
Alternative	1,463	4,061	2,480	2,063	3,182	2,491
Skewness Test	-1,772	0,228	-0,132	-0,895	0,000	-0,263
Er. std. Skewness	0,564	0,637	0,441	0,564	0,637	0,441
Minimum	18	18	18	18	18	18
Maximum	21	24	24	21	24	24
Percentiles 25	21,00	18,50	20,25	18,00	18,50	18,00
50	21,00	21,00	21,00	21,00	21,00	21,00
75	21,00	21,00	21,00	21,00	21,00	21,00
a) p<0,001 b) p<0,05 ns) p> 0,05						

*Slope profile*: Comparative slope profile by study groups (dB) (**Table 4**):

- in the study group, the slope profile in the RE varied from 1,1 to 4,5, the average level being 2,588 ± 1,006, close to the median value 2,45, and the result of the Skewness test = 0.564;

- in the study group, the slope profile in the LE varied from 1 to 4,5, the average level being 2,894 ± 1,256, close to the median value 2,750, and the result of the Skewness test = 0.564;

- in the control group, the slope profile in the RE varied from 2,4 to 3,3, the average level being 2,908 ± 0,303, close to the median value 2,800, and the result of the Skewness test = 0.637;

- in the control group, the slope profile in the LE varied from 1,8 to 3,6, the average level being 2,725 ± 0,666, close to the median value 2,750, and the result of the Skewness test = 0,637;

**Table 4:** Comparative slope profile by study groups (dB)

	RE slope profile			LE slope profile		
	Study Group	Control Lot	All groups	Study Group	Control Lot	All groups
N	16	12	28	16	12	28
Average	2,588	2,908 ns)	2,725	2,894	2,725 ns)	2,821
Median	2,450	2,800	2,750	2,750	2,750	2,750
Std. Dev	1,060	0,303	0,829	1,256	0,666	1,032
Alternative	1,124	0,092	0,688	1,577	0,444	1,064
Skewness Test	0,521	0,046	0,125	0,160	-0,119	0,275
Er. std. Skewness	0,564	0,637	0,441	0,564	0,637	0,441
Minimum	1,1	2,4	1,1	1,0	1,8	1,0
Maximum	4,5	3,3	4,5	4,5	3,3	4,5
Percentiles 25	1,700	2,700	2,100	1,750	2,100	1,950
50	2,450	2,800	2,750	2,750	2,750	2,750
75	3,375	3,250	3,300	4,475	3,425	3,500
a) p<0,001 b) p<0,05 ns) p> 0,05						

*Right ear PTA with RE: MD function, Cal HOV level, slope profile*

**Fig. 5:**
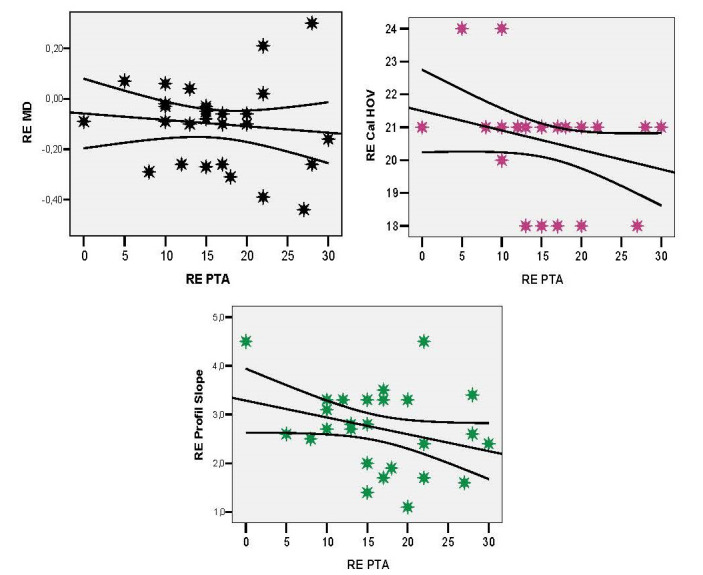
Right ear PTA correlations with the RE parameters: MD, Cal HOV level, slope profile

The correlation of the right ear PTA was indirect, reduced in intensity, both with MD (r = -0.108; p = 0.585), Cal HOV (r = -0.268; p = 0.168), or slope profile (r = -0.297; p = 0,1) (**Fig. 5**).

*Left ear PTA with LE parameters: MD function, Cal HOV level, slope profile*

**Fig. 6:**
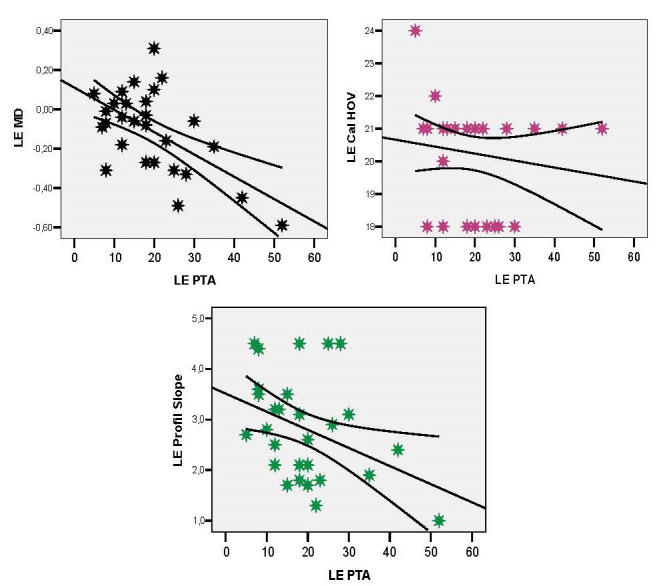
Left ear PTA correlations with the LE parameters: MD, Cal HOV level, slope profile

The left ear PTA correlation was indirect, moderate in intensity, statistically significant with both MD (r = -0.584; p = 0.001) and slope profile (r = -0.377; p = 0.048) and reduced as intensity with Cal HOV (r = -0.147; p = 0.456) (**Fig. 6**).

## Discussions

The cognitive and functional decline of the body were associated with the loss of hearing and vision, but also with the dual impairment (both hearing and vision deficiency) [**18**]. Being two extremely important elements in the functioning of the body, the two visual and auditory systems have generated numerous studies both from an ophthalmological and auditory point of view. It should be mentioned that in most of the studies, the visual function was evaluated only in terms of the evolution of visual acuity. In the study conducted, we assessed the evaluation of the visual function by analyzing the parameters of the visual field like a priority, because it demonstrated that the peripheral perimetric damage in glaucoma was the most important influence on the quality of life, knowing the cases with considerable peripheral perimetric deficits and preserved central visual acuity [**18**]. An increased share of female patients (81.4%), compared to men (28.6%), resulted from the demographic analysis of the patients in this study. The statistical analysis showed a high percentage of patients with above-average education (high school, university 71.4%) in the studied groups. As a result, it could be affirmed that the female patients were more compliant and that the studies above the average made it easier for them to accept to participate in the study. Also, the increased number of functional investigations (VF; PTA) and brain MRI have further reduced the acceptance rate beyond the inclusion criteria, which were quite restrictive, and justified the low number of participants included in the study. Analyzing the area of origin, it was found that the majority was the percentage of those from the urban environment compared to those from the rural environment (82.1% versus 17.9%). Each patient’s expertise had to be obtained through some functional tests, and the cognitive ability acquired through experience and own exercise had a key role. As a first conclusion deriving from the primary demographic analysis, it could be affirmed that women presented to the doctor more quickly and more frequently, and that the urban origin combined with the level of schooling made it easier to understand the performance of the functional tests that required a considerable amount of understanding and completion from the patients.

The evaluation of the results obtained from the analyses of the clinical parameters showed variations in the VF parameters expected in the glaucomatous diseases. The correlations between the functional evaluation of the optic nerve and the auditory were quite interesting. Following the statistical analysis, it was observed that there was an indirect correlation in the RE, reduced in intensity between the VF parameters: MD (r = -0.108; p = 0.585), Cal HOV (r = -0.268; p = 0.168) and slope profile (r = -0.297; p = 0.1), and right ear PTA. In the LE, the parameters MD (r = -0.584; p = 0.001) and slope profile (r = -0.377; p = 0.048) correlated indirectly, moderately, but statistically significant with the left ear PTA and less with Cal HOV (r = -0.147; p = 0.456). The observed correlations could be discussed if we analyzed the embryological origin, the anatomy and physiology of the visual and auditory pathways and the somatotopic distribution of the optic nerve fibers. From an embryological point of view, the optic and auditory nerves have a common embryological origin from the neuroectoderm. If we analyzed the embryological evolution of the two systems, we would observe the following:

- The inner ear, which contains the organ of Corti that translates acoustic signals into neural impulses, derives from the ectoderm and is the first of the three anatomical parts of the ear. The development begins on the ectoderm surface, because the otic placode or otic disc is one of the first sensory placodes involved in the development of the special sensory organs [**19**].

- The visual analyzer starts growing in the 3rd week of gestation with the optic grooves of neuroectodermal origin in the forebrain [**20**,**21**]. The optic vesicle develops from the optic grooves and optic stalk will develop from the optic vesicle (the future optic nerve), and will generate the necessary modifications in the ectoderm surface to develop the placode lens. At four weeks of gestation, the optic vesicle invaginates and creates the optic cup [**22**], which will become the retina.

From the view point of the functional anatomy of the visual and auditory pathways, several elements that emphasize the central connection of the visual and auditory analyzers are important.

The light information transformed into nerve signals leaves the retina through the optic nerves made up of ganglion cell axons. At the level of the optic chiasm, located near the pituitary gland, the fibers of the optic nerve from the nasal part cross and continue their path along with the fibers that collect information from the temporal part of the retina of the contralateral eye to the lateral dorsal geniculate nuclei and later form the geniculocalcarine tract, while the optic radiations reach the primary visual cortex located on the edges of the calcarine sulcus, in the medial area of the occipital lobe. Neural circuits responsible for receiving related impulses, processing information and generating other efferent responses are present in the central nervous system (CNS). A key role is played by neuronal patterns [**23**], because the neurons cannot function alone, although in complex organisms, neurons specialized for various functions are present. In complex organisms, the CNS can create types of behaviors that recognize subsystems and neural pathways that include sensory afferents coming from the retina, but considering that each specific cortical region is interconnected in both directions with other regions that ensure the primary functions of the motor example, the cortical integration of visual information becomes a complex mechanism closely connected with other motor, auditory, and vestibular cortical areas [**23**]. 

The primary somatosensory areas receive afferents from the visual and auditory analyzers and play the role of detecting specific visual and auditory sensations. The secondary somatosensory areas decipher the sense of the impulses reaching the primary areas. They generate their interpretation, initiate the evaluation of specific sensory impulses that include: the understanding of the shape or texture of an object, the analysis of colors, light intensity, the direction of lines, angles and different aspects of visual perception, the interpretation of tones, their intensity and the sequence of tones contained by auditory nerve impulses. Association areas, which receive and analyze impulses from several areas of the motor and sensory cortex, as well as from the subcortical areas, are also present at the cortical level, besides the primary and secondary or somatosensory areas. The significant association areas are the following: parieto-occipital-temporal, prefrontal and limbic. The parieto-occipital-temporal association area is situated at the level of the parietal and occipital cortex, being delimited anteriorly by the somatosensory cortex, posteriorly by the visual cortex, and laterally by the auditory cortex. At the level of this area, the sense of the impulses coming from all the nearby somatosensory areas are interpreted [**15**,**23**,**24**].

Considering all the complex interrelations between the primary, secondary and cortical association areas, in which visual and auditory information are processed, the connections between the changes in the function of the optic and auditory nerve become possible. Neuroconnectivity of the brain can explain many of the connections between the visual and auditory analyzer.

## Limitations

Unfortunately, the study had a series of limitations that must be mentioned: low number of patients included, the fact that the study was monocentric and had a cross-sectional nature, short follow-up period, severe inclusion criteria that generated few enrollments. Also, patients with mild glaucomatous damage were enrolled in our study, and starting from this point, it would be interesting to develop further studies considering groups with different glaucomatous damage, but which would allow the performing of functional evaluations.

## Conclusions

The observations were interesting, challenging and opened opportunities for further study. The association of changes in VF parameters, light sensitivity at 10° (Cal HOV) and the profile of the decrease in light sensitivity from the core to the periphery (slope profile), with hearing values on the same side, could be explained from an embryological, anatomical and physiological evolution, if the visual and auditory pathway was analyzed functionally and structurally. But, due to the limitations of the study, the final conclusions could not be drawn. These changes and associations must be noticed within a general context, together with the evaluation of the structure of the optic nerve and its changes in glaucoma, while opening new opportunities and approaches with a longitudinal design to observe how these parameters change over time. Ensuring the quality of life is still the main objective since the aging of the population has a global character and implies an increase in the glaucomatous disease. The multidisciplinary nature of glaucoma respects once again the idea issued in 1922 by Felix Lagrange that the eye with glaucoma is “a sick eye in a sick body”.

**Conflict of Interest statement**

The authors state no conflict of interest.

**Informed Consent and Human and Animal Rights statement**

Informed consent has been obtained from all individuals included in this study.

**Authorization for the use of human subjects**

Ethical approval: The research related to human use complies with all the relevant national regulations, institutional policies, is in accordance with the tenets of the Helsinki Declaration, and has been approved by the Ethics Committee of Clinical County Emergency Hospital of Brăila, Romania (no. 1/ 3.09.2021).

**Acknowledgements**

None.

**Sources of Funding**

None.

**Disclosures**

None.

## References

[1] Allison K, Patel D, Alabi O (2020). Epidemiology of Glaucoma: The Past, Present, and Predictions for the Future. Cureus.

[2] Wallhagen MI, Strawbridge WJ, Shema SJ, Kurata J, Kaplan GA (2001). Comparative impact of hearing and vision impairment on subsequent functioning. JAGS.

[3] Brennan M, Bally SJ (2007). Psychosocial adaptations to dual sensory loss in middle and late adulthood. Trends Amplif.

[4] Fischer ME, Cruickshanks KJ, Klein BEK, Klein R, Schubert CR, Wiley TL (2009). Multiple sensory impairment and quality of life. Ophthalmic Epidemiology.

[5] Crews JE, Campbell VA (2004). Vision impairment and hearing loss among community-dwelling Americans: Implications for health and functioning. Am J Public Health.

[6] Brennan M, Horowitz A, Su YP (2005). Dual sensory loss and its impact on everyday competence. Gerontologist.

[7] Chia EM, Mitchell P, Rochtchina E, Foran S, Golding M, Wang JJ (2006). Association between vision and hearing impairment and their combined effects on quality of life. Arch Ophthalmol.

[8] Capella-McDonnall ME (2005). The effects of single and dual sensory loss on symptoms of depression in the elderly. Int J Geriatr Psychiatry.

[9] Lin MY, Gutierrez PR, Stone KL (2004). Vision impairment and combined vision and hearing impairment predict cognitive and functional decline in older women. J Am Geriatr Soc.

[10] Schneck M, Lott L, Haegerstrom-Portnoy G, Brabyn J (2012). Association between hearing and vision impairments in older adults. Ophthalmic Physiol Opt.

[11] Popa-Cherecheanu A (2019). Ghid practic de perimetrie computerizata. Cap. II.

[12] (2016). Optopol PTS 1000 Automated Perimeter Instruction manual. ver. 4.5, rev A, Chap. 10.

[13] Walker JJ, Cleveland LM, Davis JL, Seales JS (2013). Audiometry screening and interpretation. Am Fam Physician.

[14] Salmon M, Brant J, Hohman M, Leibowitz D (2023). Audiogram Interpretation.

[15] (2017). Guyton & Hall. Tratat de fiziologia omului. Capitolul 50. Analizatorul vizual II: Functia de receptor si functia neurala a retinei, Capitolul 51. Analizatorul vizual III. Neurofiziologia centrala a vederii, Capitolul 52. Analizatorul auditiv, Capitolul 57. Cortexul cerebral, functiile intelectuale ale creierului invatarea si memoria.

[16] Dobie RA, Van Hemel S (2004). National Research Council (US) Committee on Disability Determination for Individuals with Hearing Impairments. Hearing Loss: Determining Eligibility for Social Security Benefits.

[17] (2023). ASHA recommendations online: https://www.asha.org.public/hearing/Type, Degree, and Configuration of Hearing Loss.

[18] Mudie L, Varadaraj V, Gajwani P, Munoz B, Ramulu P, Lin F, Bonnielin KS, Friedman DS, Nazlee Z (2018). Dual sensory impairment: The association between glaucomatous vision loss and hearing impairment and function Hearing and vision impairment: Effect on function. PLOS ONE.

[19] Helwany M, Tafline C, Arbor T, Tadi P (2023). Embryology Ear.

[20] Edward DP, Kaufman LM (2003). Anatomy, development, and physiology of the visual system. Pediatr Clin North Am.

[21] Bales T, Lopez M, Clark J (2023). Embryology.

[22] Heavner W, Pevny L (2012). Eye development and retinogenesis. Cold Spring Harb Perspect Biol.

[23] Boron W, Boulpaep E (2017). Fiziologie medicala. Sectiune III, cap. 15 Transductia senzoriala, Cap 16. Circuite neuronale.

[24] Cernea P (2002). Tratat de Oftalmologie. Cap. 14. Retina, Histologie.

